# Dislokation eines STARflo-Glaukomdrainageimplantats mit begleitenden Komplikationen

**DOI:** 10.1007/s00347-021-01382-9

**Published:** 2021-04-21

**Authors:** S. Schrittenlocher, A. Doulis, K. R. Koch, A. Lappas, L. Altay, A.-M. Lentzsch, C. Cursiefen, B. Bachmann, V. Prokosch-Willing, T. Dietlein

**Affiliations:** Medizinische Fakultät und Uniklinik Köln, Zentrum für Augenheilkunde, Universität zu Köln, Köln, Deutschland

## Falldarstellung

### Anamnese und klinischer Befund

Ein 62-jähriger männlicher Patient mit primärem chronischem Offenwinkelglaukom wurde am Zentrum für Augenheilkunde der Universität zu Köln aufgrund zunehmender Sehverschlechterung und Fremdkörpergefühl seit einer extern durchgeführten STARflo™-Implantation (iStar Medical, Wavre, Belgien) und Phakoemulsifikation mit Implantation einer Hinterkammerlinse am rechten Auge vor 3 Jahren vorgestellt. Das Auge war vor der kombinierten Operation mehrfach extern voroperiert worden (selektive Lasertrabekuloplastik, Goniotrepanation mit Mitomycin C und Zyklophotokoagulation). Die Therapie bestand aus einer lokalen und systemischen drucksenkenden Therapie.

**Figure 1:**
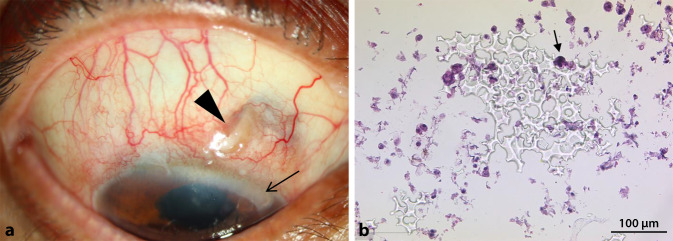

Bei der klinischen Untersuchung in unserer Augenklinik war der Visus RA cc (+7,75/−0,75 × 49) = Handbewegung und LA cc (+1,25/−1,0 × 99) = 0,1 und der Augeninnendruck (IOD) 7/20 mm Hg. Es zeigten sich rechts eine stark injizierte Bindehaut und eine perforierte Bindehaut mit Exposition des STARflo™-Implantats im nasal oberen Quadranten (Abb. 1a). Zudem zeigte sich eine Vorderkammerreizung, die Pupille spielte nicht, im Glaskörper fanden sich massiv Schlieren, die Hinterkammerlinse war in loco mit deutlichem Nachstar. Am linken Auge zeigten sich ein reizfreier Befund und eine Cataracta provecta. Funduskopisch bestand rechts bei ausgeprägtem Nachstar und Hornhautödem kein Einblick, und links zeigte sich eine randscharfe und blasse Papille. Bei eingeschränkter Bildqualität konnte man in der Makula-OCT (optische Kohärenztomographie) Aufnahme Aderhautfalten und ein Makulaödem am rechten Auge erkennen. Es wurde eine dringende operative Versorgung geplant.

## Diagnose

Bindehautperforation und Exposition eines intra-/subskleralen STARflo-Glaukomdrainageimplantats mit begleitender Uveitis.

## Therapie und Verlauf

Es erfolgte die Durchführung einer Teilexzision des Implantates mit anschließender Defektdeckung mit einem Ologenimplantat. Histologisch zeigte sich anschließend eine entzündungsfreie Mikroporenstruktur des Implantates mit einzelnen Makrophagen und mehrkernigen Riesenzellen (Abb. 1b). Postoperativ lag der IOD bei 21/7 mm Hg.

Zwei Monate später stellte sich der Patient erneut notfallmäßig mit Verdacht auf Sickerkisseninfektion und starken Schmerzen vor. Hierbei zeigte sich eine offene Bindehaut mit einem exponierten Ologenimplantat. Der Patient wurde zur intensivierten lokalen antibiotischen und antientzündlichen Therapie stationär aufgenommen. Darunter besserten sich Beschwerden und der Entzündungsreiz, sodass zunächst auf eine Revision verzichtet wurde.

Nach 2 weiteren Monaten zeigten sich am rechten Auge ein Bindehautdefekt mit exponiertem Implantat und hintere Synechien. Der IOD betrug 21/26 mm Hg. Am darauffolgenden Tag erfolgte eine erneute Revision. Intraoperativ wurde das Ologenimplantat zum Teil reseziert. Es konnte eine Skleromalazie festgestellt werden, sodass eine Deckung mit gewässerter Sklera erfolgte. Darüber wurde ein freies Bindehauttransplantat aus dem unteren Fornix fixiert. Der IOD am rechten Auge schwankte postoperativ zwischen 13 und 28 mm Hg.

**Figure 2:**
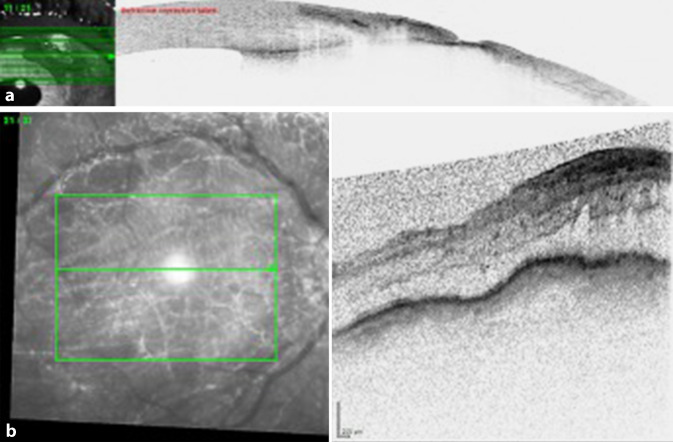

**Figure 3:**
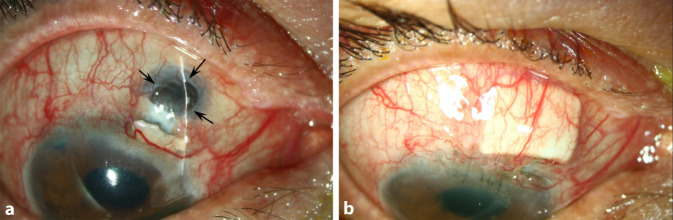

Ein Jahr nach der ursprünglichen Vorstellung wurde der Patient erneut wegen eines Skleradefekts mit exponiertem, aber konjunktivalisiertem Uveaareal stationär aufgenommen. Der IOD lag bei 16/20 mm Hg. Der Visus war RA = Handbewegung und LA (−1,5/−0,5 × 22) = 0,05. In der Vorderkammer zeigten sich am rechten Auge frische Endothelbeschläge, Zellen 1+ und Tyndall. Die Pupille war komplett synechiert. Die Vorderkammer zeigte sich tief, das Seidel-Phänomen war positiv. In der Vorderabschnitts-OCT zeigte sich eine Skleromalazie (Abb. 2a). Links war der Befund reizfrei. In der Makula-OCT zeigte sich ein massives zystoides Makulaödem (Abb. 2b), das bereits seit mindestens einem Jahr bestand. Angiographisch zeigten sich eine gestörte Blut-Retina-Schranke und Hypotoniefalten. Ischämische Zeichen konnten ausgeschlossen werden. Ein Uveitissuchprogramm zeigte sich nicht wegweisend. Es erfolgte eine erneute operative Versorgung am kommenden Tag. Intraoperativ wurde das restliche STARflo-Implantat ab externo entfernt, und ein Sklerapatch wurde zur Defektdeckung eingenäht. Die Abb. 3a, b zeigt den Befund prä- und postoperativ.

Die Beschwerden des Patienten waren deutlich rückläufig, und der IOD schwankte zwischen 17 und 28 mm Hg. Bei Entlassung zeigte sich ein terminentsprechender Befund, der IOD lag bei 17/22 mm Hg. Das Hornhautödem und das zystoide Makulaödem waren rückläufig. Bei der letzten ambulanten Kontrolle betrug der IOD 14/14 mm Hg unter 3‑facher drucksenkender Lokaltherapie, und der Sklerapatch zeigte sich reizfrei und in loco. Der Patient wurde der weiteren ambulanten Behandlung des niedergelassenen Augenarztes überlassen.

## Diskussion

Dies ist die Erstbeschreibung einer solchen komplexen Komplikation nach STARflo™-Implantation. Das STARflo™-Drainageimplantat befindet sich seit 2012 auf dem europäischen Markt (Hersteller iStar Medical, Wavre, Belgien) und gehört zu der Gruppe der BAGS (Bleb-free Ab-externo Glaucoma Surgery). Es handelt sich um ein suprachoroidales Glaukomdrainageimplantat aus einem patentierten, porösen Silikonmaterial (STAR® Materialhersteller Nusil Technologies LLC, Carpinteria, USA).

Das Implantat besteht aus 3 Anteilen: Kopf‑, Hals‑ und Körperregion und misst 8 mm in der Länge, 5 mm in der Breite und hat eine Dicke von 275 µm. Es wird ab externo über einen Deckel implantiert. Das Körperteil wird suprachoroidal platziert und der Kopfanteil wird im Kammerwinkel belassen. Dank der speziellen Porenstruktur soll ein geregelter Durchfluss an Kammerwasser gewährleistet werden.

Die größte in der Literatur vorbeschriebene Kohorte ist die von Fili et al., die über 36 Augen berichteten. Die Autoren schilderten 2018 frühe postoperative Komplikationen wie okuläre Hypotonie, Hyphäma, Glaskörperblutung und eine Fibrinreaktion in der Vorderkammer, die allerdings transient waren. Allgemein zeigte das Implantat eine befriedigende Drucksenkung von 28,8 % und eine Augentropfenreduktion von 66,7 % nach 1 Jahr [3]. Weiterhin berichteten andere Autoren über postoperativ transienten Astigmatismus, transiente Hypotonie und Aderhautamotio [2, 4].

Eine Perforation des STARflo™-Glaukomdrainageimplantats mit Skleraeinschmelzung und Uveitis ist in der Literatur bislang nicht beschrieben worden. In dem dargestellten Fallbericht wird eine Dislokation des STARflo™-Glaukomdrainageimplantats mit Bindehautperforation und konsekutiver Wundheilungsstörung beschrieben. Eine Skleromalazie infolge von mehreren Voroperationen wie auch nach Trabekulektomie mit Mitomycin C ist bereits bekannt [1]. Limitierend zu den möglichen Ursprüngen der Komplikationen zu erwähnen ist, dass der Patient zuvor eine Trabekulektomie erhalten hatte. Die auswärts operierte vorangegangene Filterzone war im Bereich der Iridektomie bei 11 Uhr temporal oben angelegt worden. Das STARflo-Drainageimplantat wurde zwar in dem benachbarten Quadranten, nasal oben, eingesetzt. Jedoch könnte die Applikation von MMC während der Trabekulektomie des benachbarten vorbehandelten Areals durch die veränderte Sklera und Bindehaut im Bereich des großen Skleradeckels wesentlich diesen ungünstigen Verlauf mit bedingt haben.

Das Außergewöhnliche bei diesem Fall stellten der persistierende intraokulare Reiz und das chronische Makulaödem dar. Bereits in dem ersten Makula-OCT-Bild waren bei eingeschränkter Bildqualität ein Makulaödem und Aderhautfalten zu erahnen, die am ehesten auf eine zurückliegende Hypotonie zurückzuführen sind. Die neu aufgetretene Panuveitis mit deutlichen Endothelpräzipitaten, vereinzelten Glaskörperzellen und massiver Schrankenstörung sind am ehesten als Folge der chronischen Uveaexposition zu bewerten. Differenzialdiagnostisch, unabhängig vom uveitischen Reizzustand, kann eine (transiente) okuläre Hypotonie auch ursächlich für ein chronisches Makulaödem sein.

Eine suprachoroidale Lage des Implantates prädisponiert bei Dislokation zu einem stetigen Abrieb zwischen Uvea und Implantat, der den chronischen Reiz und das Entwickeln einer Skleromalazie erklären könnte. Durch die Deckung mit einem Sklerapatch sind die Wundheilungsstörungen operativ erfolgreich zu behandeln. Eine ähnliche Situation wurde vor mehreren Jahren von Lüke et al. im Rahmen einer Viskokanalostomie mit Implantation eines hochvernetzten Hyaluronsäureimplantats (SK-GEL®) vorbeschrieben. In der Kasuistik kam es ebenfalls zu einer Implantatexposition, die allerdings ohne weitere chirurgische Maßnahmen unter alleiniger Salbentherapie abheilte [5]. Die Indikation zum Revisionseingriff hängt maßgeblich von dem klinischen Befund und vom intraokularen Reizzustand ab.

## Fazit für die Praxis

- Eine Arrosion und Dislokation durch die Bindehaut des STARflo-Glaukomdrainageimplantats scheint eine seltene, aber mögliche Komplikation trotz subskleraler Implantationslage zu sein.
- Wundheilungsstörungen und Komplikationen können im Verlauf auftreten.
